# Physiological Considerations for Modeling *in vivo* Antibody-Target Interactions

**DOI:** 10.3389/fphar.2022.856961

**Published:** 2022-02-24

**Authors:** Tyler Dunlap, Yanguang Cao

**Affiliations:** ^1^ Division of Pharmacotherapy and Experimental Therapeutics, Eshelman School of Pharmacy, University of North Carolina at Chapel Hill, Chapel Hill, NC, United States; ^2^ Lineberger Comprehensive Cancer Center, School of Medicine, University of North Carolina at Chapel Hill, Chapel Hill, NC, United States

**Keywords:** physiological factors, therapeutic antibody, target binding, receptor occupancy, MIDD, modeling and simulation

## Abstract

The number of therapeutic antibodies in development pipelines is increasing rapidly. Despite superior success rates relative to small molecules, therapeutic antibodies still face many unique development challenges. There is often a translational gap from their high target affinity and specificity to the therapeutic effects. Tissue microenvironment and physiology critically influence antibody-target interactions contributing to apparent affinity alterations and dynamic target engagement. The full potential of therapeutic antibodies will be further realized by contextualizing antibody-target interactions under physiological conditions. Here we review how local physiology such as physical stress, biological fluid, and membrane characteristics could influence antibody-target association, dissociation, and apparent affinity. These physiological factors in the early development of therapeutic antibodies are valuable toward rational antibody engineering, preclinical candidate selection, and lead optimization.

## Introduction

In 1984, the first therapeutic monoclonal antibody was approved by the U.S. Food and Drug Administration (FDA). In 2021, the 100th antibody was approved just 6 years following approval of the 50th (Mullard, 2021). This trend highlights the accelerating interest and clinical application of antibody-based therapeutics. The ability to modulate cell-surface and soluble targets with high affinity and specificity make these molecules attractive therapeutic modalities. With a phase I to approval success rate of approximately 22% (Kaplon and Reichert, 2019), nearly double that of small molecule drugs, drug developers are increasingly shifting their focus toward protein drug development (Kaplon et al., 2020). Although antibodies and small molecule drugs share similar clinical development paths, antibody-based therapeutics present unique challenges from the early stage of candidate selection to the late stage of therapeutic confirmation (Tang and Cao, 2021).

Bringing a therapeutic antibody to market requires a team of scientists across multiple disciplines closely collaborating in all stages of development. At the early stage, after the therapeutic target for an indication is selected, decisions must be made regarding the design format, affinity requirement, feasibility of efficacious doses, and candidates for subsequent stages. Rational lead optimization and candidate selection are critical tasks in early drug development and can differentiate success and failure in clinical stages. Antibody engineering provides means for controlling a candidate’s half-life, affinity, and biological activity (Chiu et al., 2019). Computational modeling and simulation can be helpful to explore these engineered parameters before comprehensive experimental evaluation and thereby provide early insights for antibody engineering. The iterative learn and confirm paradigm between antibody engineering and computational modeling exemplifies model informed drug development (MIDD) in preclinical drug development, which seeks to leverage mathematical and statistical models to optimize drug development processes. In the preclinical stage, one critical MIDD task is to evaluate plausible ranges of target binding affinity and clinically feasible doses likely to achieve adequate target engagement.

Antibody-target interactions take place within specific tissue environments with characteristic physiological attributes. The physiology of these local environment critically influences antibody-target interactions resulting in apparent affinity alterations and heterogenous target engagement. Contextualizing these *in vivo* interactions by integrating local physiological factors beyond those commonly considered in physiologically based pharmacokinetic (PBPK) models could enhance model prediction fidelity and boost confidence in early-stage decisions (Cao et al., 2013; Cao and Jusko, 2014). For instance, if the binding rate between antibody and target is high, association and dissociation are primarily restricted by the diffusion rate of antibody to or away from the target in the local tissue and cellular environment. In this case, the apparent rate of association and dissociation will become context-dependent, not directly reflective of the intrinsic reaction rate. Incorporating this kind of physiological intuition into early-stage models depicting antibody-target interactions could yield insights toward optimal antibody design and affinity thresholds. MIDD approaches should leverage knowledge of tissue microenvironment and local physiology to guide preclinical candidate selection, antibody design, and lead optimization. Here we briefly review how physiological factors can influence antibody-target engagement and demonstrate these concepts toward optimizing preclinical decision-making processes.

## Antibody-Target Interactions

### Antibody-Target Affinity: *In Vitro* Approaches and Problems

Surface plasmon resonance (SPR) is a label-free technique to measure the kinetics of molecular interactions and has become the standard for *in vitro* characterization of antibody-target binding (Olaru et al., 2015). An extension of this technology is SPR imaging which directly measures cell surface antibody-antigen binding kinetics and can be used to estimate binding affinity and antigen density (Zhang et al., 2020). Major advantages to this technique are that interacting species need not be labeled and binding events can be visualized in real-time, allowing for measurement of association and dissociation rates. Inherent problems to this method include mass transport limitations and surface site heterogeneity. Strategies for analyzing SPR data to account for these complexities are reviewed elsewhere (Schuck and Zhao, 2010). In addition, flow cytometry has also become an approach applied to assess antibody-target engagement in blood cells and tissue-derived cell samples (Moulard and Ozoux, 2016).

The slow dissociation rate of antibodies from their target necessitates relatively long incubation times to reach equilibrium compared to small molecule drugs. Equilibrium states are, by definition, invariant with time; thus, determining accurate estimates requires the demonstration of negligible change in product and reactant amounts over time. For therapeutic antibodies with pM or nM affinities, it takes hours, even days, to reach binding equilibrium with their targets. However, nearly 90% of reported incubation times for equilibrium constants in a survey by Jarmoskaite et al. were an hour or less (Jarmoskaite et al., 2020). Jarmoskaite et al. provide two recommendations for establishing confidence in reported equilibrium constants, publishing the time to equilibrium and demonstrating that the dissociation constant is not susceptible to titration (Jarmoskaite et al., 2020). Furthermore, while a single equilibrium constant is often reported for ligand-receptor interactions, association and dissociation are concentration- and context-dependent (Berkers et al., 1992). Individual equilibrium estimates likely reflect the mean of a distribution of experimental values calculated for a given reaction (Reverbi and Reverbi, 2007). The inherent uncertainty in reported values warrant careful consideration when using published rate constants in models depicting target engagement, as commonly done in pharmacokinetic (PK) and pharmacodynamic (PD) models. Reporting statistical metrics, such as the standard error or coefficient of variation of parameter estimates derived from experimental data should be encouraged and may promote greater appreciation for the uncertainty in calculated rate constants.

**FIGURE 1:**
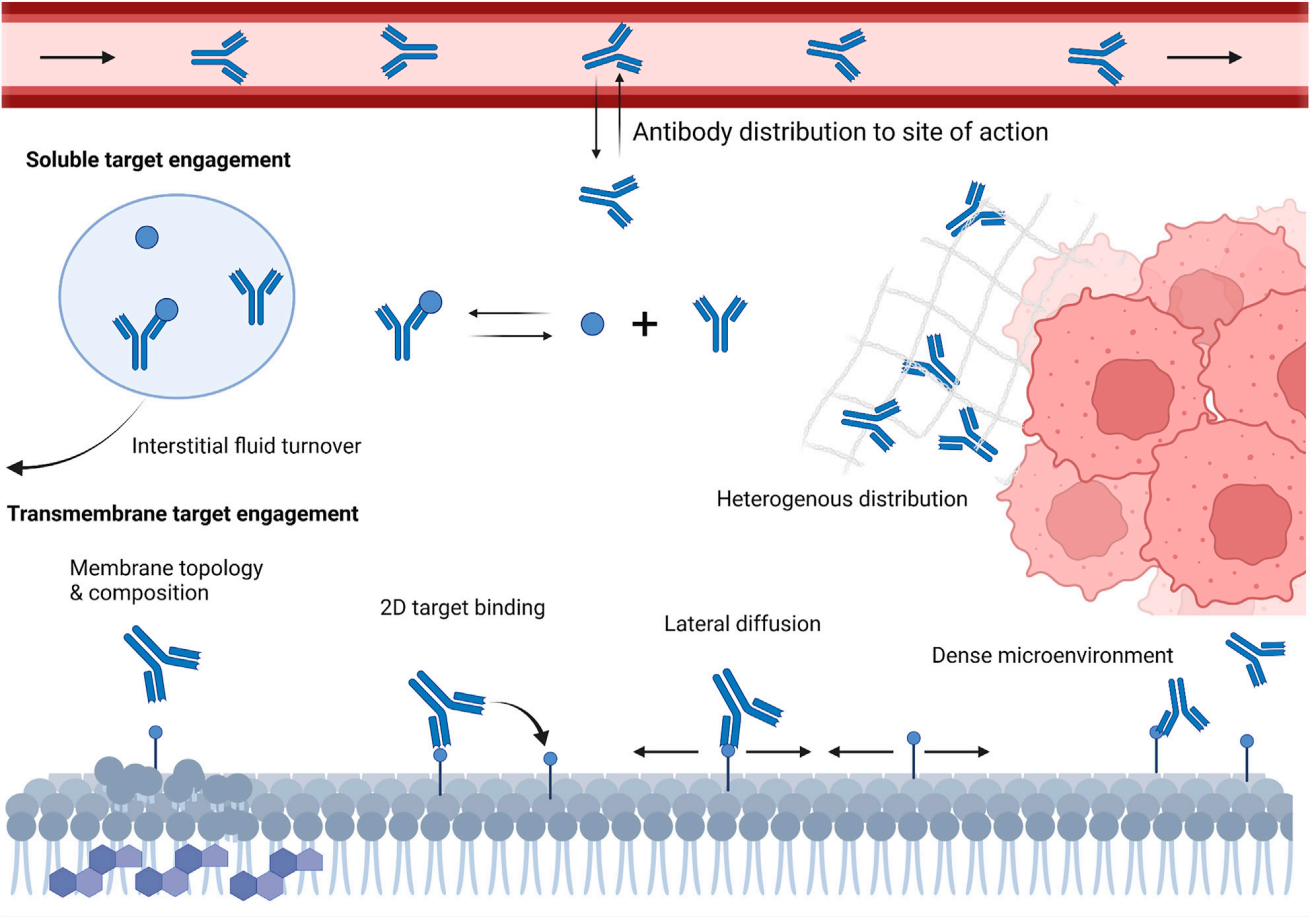
Schematic representation of various local microenvironment and physiological factors that can influence antibody-target engagement *in vivo*. These factors include antibody slow and poor distribution into target tissues, interstitial fluid turnover, restricted antibody diffusion in dense microenvironment, cell membrane topology and composition, and target density and lateral diffusivity.

Equilibrium rate constants (e.g., K_D_) are used, in essence, to summarize ligand-receptor engagement. While an affinity summary metric is theoretically useful, understanding both the association (k_on_), and dissociation (k_off_) rates are essential for *in vivo* characterization of antibody-target interactions. These rate constants describe the microkinetic relationship between individual antibody domains and corresponding binding domains on the target. The “intrinsic” value to these rate constants may be estimated through *in vitro* techniques, such as SPR. However, these values do not reflect binding under physiological conditions. Understanding antibody-target interactions under physiological conditions is necessary for developing accurate foresight into the potential efficacy of preclinical antibody candidates; yet remains largely uncharacterized at the very early stage. A schematic representation of concepts depicting how the microenvironment and local physiology can influence antibody-target interactions is shown in Figure 1.

### Factors Affecting Antibody Avidity

In solution, the probability of antibody-target interactions is largely dependent upon the relative concentrations and diffusion rates of the two species (Arrhenius, 1889): with a theoretical upper limit around 10^9^ Mol^−1^s^−1^ in the absence of steric hindrance. The probability of complex formation is also influenced by bond activation energies and the orientation of the species when they encounter (Wigner, 1932; Eyring, 1935). The k_on_ and k_off_ essentially summarize this information and characterize the likelihood of complex formation following physical interaction and the stability of bonds formed, respectively. Engineered increases to k_on_ (faster association rates) result in faster complex formation while decreases to k_off_ (slower off-binding rates) result in a more stable complex. The ratio between these rates (k_off_/k_on_) is used to describe the overall affinity (i.e., K_D_) of the interaction. Notably, affinity is determined by the relative difference in these rate constants, not by their individual magnitudes. While affinity can be easily estimated from *in vitro* techniques, *in vivo* affinities can be profoundly different (Fujimori et al., 1989; Sharkey et al., 1990; Shockley et al., 1992). Antibody-target interactions demonstrate that cooperativity, dimensionality, multivalency, diffusivity, and local physiology contribute to the overall affinity of an interaction. These phenomena orchestrate complex engagement dynamics that are difficult to recapitulate *in vitro*. Avidity, also referred to as “functional affinity”, depicts the affinity of two species in their native environment by accounting for synergistic/antagonistic physical properties and environmental influences (Erlendsson and Teilum, 2020). Avidity is conceptually intuitive but challenging to predict. There is no single quantity that can be described as “the avidity” of an interaction because the value is context-dependent (Kaufman and Jain, 1992). Discrepancies between *in vitro* affinity and *in vivo* avidity highlight the poorly understood influence of the microenvironment on molecular interactions.

An often-overlooked factor influencing the avidity of an interaction is the geometry of the physiological space in which the species interact (Coombs and Goldstein, 2004). Cells and tissues are highly compartmentalized ecosystems often spatially restricted across multiple dimensions. This dimensionality reduction can influence the k_on_ and k_off_ of an interaction in many ways. For example, diffusion of antibodies adhered to the cell membrane and transmembrane targets are confined to two dimensions (i.e., laterally on the cell surface) and further restricted by other macromolecules, membrane rigidity, tight junctions, etc. The diffusion rate of bound antibodies on lymphocytes has been estimated to be approximately 10^–10^ cm^2^/s (Elson et al., 1976), nearly four orders of magnitude lower than the diffusion constant in solution. These hindrances influence the avidity between two species by reducing molecular dispersion on the cell surface after dissociation events, thereby promoting rebinding (Mosquera et al., 2020). Rebinding can contribute to apparent increases in k_on_, or decreases in k_off_, between molecules *in vivo* (Vauquelin and Charlton, 2013). Coombs and Goldstein propose the effects of hindered diffusion (i.e., diffusion through a dense microenvironment) can be approximated by calculating a modulated rate constant considering the compartment dimensions, flux, and diffusion coefficients of the interacting species (Coombs and Goldstein, 2004).

### Target Engagement and Antibody Efficacy Metrics

A critical element to PD theory is the receptor occupancy model (Hill, 1909), based on the premise that receptor engagement will translate to modulation of downstream biology and that PD effect is closely related to the fraction of receptors engaged. Models extending receptor occupancy to account for complex signaling phenomena such as fractional occupancy, constitutive activity, and nonlinear transduction have been developed to further appreciate the complexity of drug action (Buchwald, 2019). Widely accepted models depicting various antibody PD mechanisms remain largely undeveloped.

A variety of antibody formats can be used to bind targets, block signaling, stimulate receptor internalization/degradation, deliver cytotoxic payloads, and more. Given this mechanistic diversity, the traditional implementation of receptor occupancy theory may be inappropriate for antibody efficacy assessment. Oftentimes, efficacy could be driven by maximizing the number of antibody-target complexes, minimizing free target levels, or maximizing bivalent bound antibodies. For antibodies that work through antibody-dependent cellular cytotoxicity (ADCC), efficacy may not be directly related to the fraction of receptors engaged, but rather to the successful initiation of subsequent effector mechanisms (Meyer et al., 2014; Wang et al., 2015; Weiskopf and Weissman, 2015). Rituximab is one such example where the density and persistence of antibody-target complex may be more therapeutically relevant than the fraction of targets engaged (Cragg et al., 2003; Maloney, 2005; Rouge et al., 2020). Bivalent bound rituximab to its CD20 target is related to a stronger ADCC effect than monovalent bound antibodies (Cragg et al., 2003). Conversely, minimum target concentration, regardless of complex abundance, is a reliable predictor of drug effect for antibodies, like infliximab, working through neutralization of soluble antigens (Tracey et al., 2008). Similarly, for antibody-drug conjugates, intracellular delivery of payload through endocytosis is most relevant to therapeutic effect (Birrer et al., 2019). We should differentiate antibodies acting as agonists versus antagonists. The therapeutic efficacy appears to be more related to target engagement for agonist antibodies than antagonists do. Generally, antibody mechanism of action and target turnovers should guide appropriate drug characteristics and dosing strategies. If target turnover within target tissues is fast (i.e., rapid production), enduring antibody concentrations to neutralize newly produced antigens, or a “C_min_” approach, with relatively frequent dosing are likely preferable for strong efficacy. If target turnover is relatively slow, additional dosing will not translate into increased efficacy once a target is engaged. Thus, a “C_max_” approach with large, infrequent doses may be sufficient.

Association and dissociation kinetics influence target engagement and, thereby, influence subsequent initiation of effector functions. Effector cell cytotoxicity can be mediated through a multitude of mechanisms including: ADCC, antibody-dependent cellular phagocytosis, or initiation of the complement cascade (Meyer et al., 2014; Wang et al., 2015; Weiskopf and Weissman, 2015; Yang et al., 2019). Importantly, effector cell engagement is related to immune complex stability, primarily determined by the ratio of antibody-antigen in the complex and avidity of individual bonds (Diebolder et al., 2014). The stoichiometry of the Fab domain target binding interaction also contributes to stable immune complex formation and is crucial for effective initiation of effector functions (Pierson et al., 2007; Tajima et al., 2011; Lux et al., 2013; Strasser et al., 2019). Furthermore, the antibody Fc domain can influence the avidity of an antibody-target interaction (Abboud et al., 2010; Bournazos et al., 2014). Additional molecular features correlated with effector cell engagement include the recognized epitope, target affinity, binding orientation, and elbow angle of the antibody (Hughes-Jones, 1977; Teeling et al., 2006; Tang et al., 2007).

The diversity in antibody mechanisms of action continues to increase with increasing use of novel design formats, such as bispecific or trispecific antibodies (Wu and Demarest, 2019). Novel design formats and increasingly complex PD warrant new approaches for quantifying antibody-target interactions. Target engagement metrics, beyond fraction of targets engaged, are needed to facilitate rational selection of preclinical antibody therapeutics (Kambayashi et al., 2019).

### Target Engagement and Antibody Spatial Distribution

Conventional PK models typically assume uniform drug distribution within a given tissue as well as proportional uptake and loss from the tissue with respect to plasma concentrations. However, antibody distribution within tissues, a process affected by transvascular permeability, local target expression, target affinity, cellular internalization, and the extracellular environment (Eikenes et al., 2004; Thurber et al., 2008a), is known to be very heterogeneous. Within tissue microenvironment, antibody diffusion is related to its size and interaction with other macromolecules and structures (Reiten et al., 2008; Cilliers et al., 2016). The diffusion coefficient of an antibody in solution, without consideration for the environmental architecture, may provide unrealistic expectations for the molecule’s ability to traverse a physiological space (Davies Cde et al., 2002). Techniques, such as fluorescence correlation spectroscopy, have been used to explore antibody diffusion and protein-protein interactions in biological matrices (Lagerkvist et al., 2001; Hung et al., 2019). Antibody diffusivity in the body can range from relatively unrestricted (e.g., in plasma) to severely hindered in densely packed physiological spaces (e.g., solid tumors and brain). When relatively unrestricted, the upper rate limit of diffusion will be rate limiting to the probability of species engagement. In these situations, increasing k_on_ beyond this theoretical rate limit will not increase the probability of species interaction. Engineering efforts should therefore focus on optimizing k_off_ to promote stable target binding.

If the target exists exclusively within densely packed physiological spaces (e.g., tumor microenvironment), only a small fraction of antibody molecules may access the area (Netti et al., 2000; Davies Cde et al., 2002). However, once accessed, the residence time of the molecule may be increased by the restrictive environment, thereby contributing to apparent increases in target affinity (Vauquelin, 2016; Tang and Cao, 2020). The density of extracellular matrices within tumor tissues can also be very heterogeneous creating a diverse landscape surrounding transmembrane targets. (Davies et al., 1997). Distinct microenvironment and high interstitial pressure, in conjunction with antibody target binding characteristics, likely contribute to the spatial heterogeneity of antibody distribution within tumor tissues (Fujimori et al., 1989; Weinstein and Van Osdol, 1992; Flessner et al., 2005; Baker et al., 2008; Tang and Cao, 2020). Accumulation and retention of cetuximab within relatively stroma-rich tissue regions has been shown even after systemic antibody has been eliminated (Tang and Cao, 2020). Heterogeneous distribution of trastuzumab, with higher levels of target-bound drug found in transverse tumor tissue, has also been observed (Baker et al., 2008).

The binding site barrier effect is a widely acknowledged concept in which high-affinity antibodies show strong perivascular distribution within tumor tissues (Fujimori et al., 1989). The theory suggests an inverse relationship between antibody-target affinity and antibody tissue penetration and may result in nonlinear PK behavior through target mediated drug disposition (Mager and Jusko., 2001). In the absence of target saturation, high target density and high affinity binding create a PK sink in which antibody diffusion through the tissue becomes significantly restricted (Weinstein and Van Osdol., 1992). This phenomenon may be exacerbated by bivalent binding, reduced dimensionality, and other factors that promote rebinding events (e.g., cross-arm binding efficiency, high k_on_, dense microenvironment). Collectively, heterogeneous antibody distribution within tumor tissues could affect treatment outcomes by promoting survival and resistance of unexposed cells.

## Antibody-Souble Target Interactions

### Biological Fluid Turnover

Targeting soluble, pathologically relevant targets (e.g., TNFα, IL-17, and IL-1β) have been a common strategy for therapeutic antibodies, particularly for treating autoimmune diseases (Hafeez et al., 2018). Soluble targets may exist in the circulation or be largely confined to a pathologically relevant tissue compartment. An important consideration for these targets is the turnover rate of biological fluid within the tissues. Interstitial fluid (ISF) turnover is the efficiency of lymphatic drainage in tissues. Significant variability in ISF turnovers have been shown between tissue types, and the physiological processes underpinning this turnover may be affected by diseases (Petrova and Koh, 2020). ISF turnover affects antibody-target engagement, binding equilibrium, as well as antibody-target complex accumulation (Li et al., 2018). The influence of ISF turnover on target binding kinetics for varying antibodies have been demonstrated previously (Li et al., 2018). This may explain why antibodies that bind the same target with similar affinities demonstrate different degrees of efficacy among disease states (e.g., Crohn’s Disease, Rheumatoid Arthritis, and Ankylosing Spondylitis).

**FIGURE 2:**
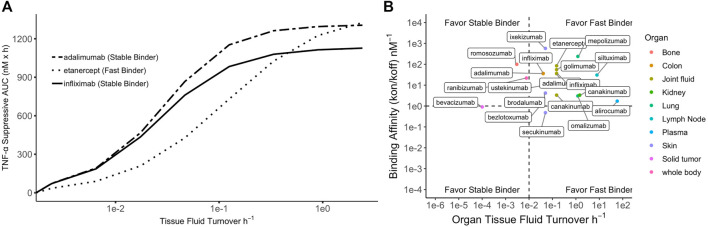
Simulated relationship between tissue fluid turnover and TNF-α suppression by three anti-TNF-α biologics. Simulation was performed using binding constants of each biologic to soluble TNF-a at their therapeutic doses. **(A)** For tissues with low fluid turnover, stable binders are favored but high affinity could contribute to accumulation of antibody-target complex; for tissues with high fluid turnover, fast binders are generally favored but lowering k_off_ beyond fluid turnover rate produces a plateau effect (i.e., affinity ceiling). **(B)** Summary of 27 licensed antibodies (Li et al., 2018) that bind soluble ligands for treatment of various diseases. Four scenarios proposed based on target binding affinity and tissue fluid turnover rate.

When ISF turnover in the diseased tissue is relatively fast, the time for antibodies and targets to engage before being washed away is limited. Maximizing target suppression through lowering k_off_ becomes increasingly challenging because rapid fluid turnover may prevent antibodies from reaching equilibrium with their targets. Tissues with higher ISF turnover rates generally experience more robust target suppression, partly due to greater antibody convections into these tissues (Li et al., 2018). Additionally, this could be, in part, attributable to the efficient removal of antibody-target complexes promoting reaction kinetics toward target suppression and complex formation. In these situations, antibodies with high k_on_ (fast binders) are preferred to promote engagement of as many targets as possible before the species are washed away. Lowering k_off_ beyond the ISF turnover may become futile, and an affinity ceiling exists. Simulations demonstrating the relative efficacy of adalimumab, etanercept, and infliximab on TNF-alpha target suppression under various ISF turnover conditions is shown in Figure 2.

When the fluid turnover rate is slow, stable binding antibodies (low k_off_) demonstrate greater target suppression than fast binders (high k_on_). However, the reaction equilibrium may shift toward dissociation due to antibody-target complex accumulation (Li et al., 2018). Soluble-target binding antibodies lead to free target suppression but also serve as a target reservoir, protecting it from endogenous degradation and potentially extending plasma half-life. Increasing complex concentrations may promote a shift in reaction equilibrium toward complex dissociation, thereby reducing target suppression efficacy (Ternant et al., 2022). In tissues with slow ISF turnover, such as solid tumors, antibody distribution is also limited due to poor convection (Thurber et al., 2008b). Low distribution and complex accumulation present dual challenges for developing therapeutic antibodies for indications associated with tissues with slow ISF turnover. This may partially explain the lack of success in developing therapeutic antibodies targeting soluble targets in solid tumors, even though several pathologically validated targets exist, such as the transforming growth factor-β (Syed, 2016). Four situations in which fast or stable binders would be preferrable for optimizing target suppression are shown in Figure 2 and are overlayed with licensed antibodies and the ISF turnover rate of their site of action.

### Antibody-CNS Target Engagement

Historically, accessing the central nervous system (CNS) with monoclonal antibodies has been challenging. Antibody concentrations in the cerebrospinal fluid (CSF) and brain ISF are typically on the order of 0.1% of plasma concentrations (Bard et al., 2012; Sevigny et al., 2016; Wang et al., 2018; Gustavsson et al., 2020). Given their poor CNS penetration, antibodies with high target affinity that elicit therapeutic effects at low concentrations are desirable for CNS targets (Tolar et al., 2020). Target selection may be limited if significant target suppression is needed for therapeutic effect. Pathological factors with low baseline levels and slow production rates may be more suitable for targeting when developing antibody therapies for CNS diseases.

Antibody-target engagement in the brain is further complicated by dynamic fluid exchange and a diverse extracellular environment (Cserr, 1988; Sykova and Nicholson, 2008; Brinker et al., 2014). Past estimates for CSF and ISF turnover in the brain are called into question due to the difficulty of measuring solute transport (Brinker et al., 2014). CSF turnover is generally greater than ISF turnover, which may have implications for antibody distribution and target engagement (Brinker et al., 2014). If the target exists predominantly in the ISF, antibody-target complex formation rate may be restricted by complex accumulation promoting reaction kinetics toward dissociation (Li et al., 2018). Furthermore, convective transport of antibodies may be constrained by the convolution of brain interstitial paths. Movement of antibodies inside the brain parenchyma has been estimated to be less than 1 mm per day (Raghavan et al., 2016). Rubenstein et al. observed that after intrathecal administration, rituximab, was cleared from the CSF slower than the rate of bulk flow (Rubenstein et al., 2003). The relatively slow convection rate of antibodies in CSF increases the chances of equilibrium bindings between antibodies and the cognate targets, which may realize the potential of antibodies with high binding affinity. The complexities of fluid transport and macromolecular diffusion within the brain remain largely unresolved.

## Antibody-Transmembrane Target Interactions

Many therapeutic targets are transmembrane proteins (e.g., EGFR, HER-2, and PD-1). These molecules face spatial and diffusional constraints, unlike soluble targets (Elson et al., 1976; Bell, 1978). The binding intricacies of transmembrane targets create engagement dynamics across time and space significantly more complex than soluble targets, particularly for bivalent antibodies. Bivalent antibody binding to a transmembrane target is a complex, two-step process that cannot be viewed as two independent monovalent steps. For example, dissociation of a bivalently bound antibody is not a first-order process, like monovalent binding. Mathematical modeling of bivalent binding at the cell surface is further complicated by dimensionality reductions, macromolecular diffusivity, antibody cross-arm binding efficiency (Kareva et al., 2018), and cell membrane characteristics.

### Cell Line Considerations

An essential consideration for any preclinical model is the validity of the cell line as a representative system for the cell/tissue of interest. This is particularly important in oncology, where each cancer cell phenotype deviates from normal host cells. Association rates between specific antibody-transmembrane targets can vary substantially between cell lines for therapeutically relevant targets, such as human epidermal growth factor receptor 2 (HER2) and epidermal growth factor receptor (EGFR), by up to an order of magnitude (Bjorkelund et al., 2011; Barta et al., 2012). The disparity in binding characteristics between cell lines may be due to differences in cell surface topology causing variability in k_on_ and k_off_ between antibody and target (Hu et al., 2013). Molecular dynamic simulations suggest the affinity of two-dimensional binding may be inversely related to the relative roughness of the cell surface, presumably due to nanoscale fluctuations in membrane shape causing macromolecular repulsion between the ligand and membrane (Hu et al., 2013). In addition to receptor expression, cell pathophysiology can influence membrane composition and contribute to heterogeneous binding (Nagy et al., 2002; Pereira et al., 2018; Zhang et al., 2019). Notably, increased cholesterol content of some breast cancer cell lines has been shown to decrease membrane fluidity and alter HER2 cell surface distribution and internalization rate (Zhang et al., 2019). When macromolecular diffusion rates at the cell surface are small, as for receptors in a viscous membrane, apparent association and dissociation rates will be reduced (Bell, 1978).

### Antibody-Transmembrane Target Binding

When an antibody binds a transmembrane target, the molecule becomes anchored to the membrane creating an effective local concentration of antibody at the cell surface. This regional concentration promotes subsequent interaction with additional targets on the cell surface (Kaufman and Jain, 1992; Pluckthon and Pack., 1997; Kramer and Karpen., 1998; Sengers et al., 2016). This local interaction between antibody-target complex and free target increases the apparent affinity of the interaction and promotes ligand rebinding. Rebinding refers to the propensity for an antibody/antibody-target complex and target to re-associate after dissociation and can contribute to significant affinity alterations, particularly for membrane-bound targets. Rebinding is a highly localized process and can refer to the re-association of primary targets or secondary target binding. In the example above, the apparent association rate of secondary binding events is increased by forced proximity of the target and the free binding arm of the antibody (Sengers et al., 2016). These secondary rebinding events are also related to antibody cross-arm binding efficiency, which measures an antibody free arm’s ability to engage targets at suboptimal binding distances (Harms et al., 2014). After initial target binding, the monovalent complex free arm undergoes a dynamic search process for a free target that becomes increasingly operative with greater hinge flexibility and decreasing molecular size. Hinge flexibility governs the arm’s propensity to engage targets at suboptimal binding distances, while the size of the molecule contributes to steric interactions and diffusivity on the membrane (De Michele et al., 2016; Sengers et al., 2016). Inclusion of a cross-arm binding efficiency parameter in preclinical models incorporates two phenomena: antibody-target complex adhered to the cell surface are restricted to a quasi-two-dimensional space, and free-arm binding is limited by rotational, torsional, and bending freedom of the antibody hinge region (Harms et al., 2014). This parameter has been suggested to be useful for rational selection of preclinical candidates (Harms et al., 2014).

Translational and rotational diffusion in two dimensions has been shown to differ greatly compared to three-dimensional diffusion (Saffman and Delbruck, 1975), thus in the event of dissociation, the two species are likely to interact again. In densely packed tissues, such as tumor microenvironments, diffusion of dissociated antibodies away from the target on the cell surface can be inhibited, promoting primary antibody rebinding events (Vauquelin, 2016). The surrounding extracellular matrix may similarly influence k_on_ and k_off_ (Morgan et al., 1998). Hindering the free three-dimensional diffusion of antibodies away from the cell surface results in prolonged “apparent” target occupancy and rebinding propensity may be directly related to the k_on_ of the interaction (Vauquelin, 2016). In microenvironment that promote target rebinding, increasing k_on_ can influence target occupancy similarly to decreasing k_off_, providing increased flexibility for antibody engineering strategies. Historically, structural antibody engineering to increase k_on_ has been more challenging relative to reducing k_off_. Although difficult, structural modifications to both the antibody-target binding domain and non-binding regions have been used to increase the association rate of antibodies or peptides to their target by order of magnitude or more (Fukunaga and Tsumoto., 2013; Muguruma et al., 2019).

## Importance of Monovalent-Bivalent Binding Modes

### Bivalent Binding-Concentration Relationships

The predominant binding mode (i.e., monovalent vs. bivalent) is critical to overall antibody selectivity, distribution, and effector function. The relative concentrations of antibodies and targets critically influence the likelihood of bivalent binding (Zuckier et al., 2000). When target density on the cell membrane is sufficient for antibodies to engage multiple targets, the proportion of bivalently bound antibodies is likely to increase with increasing target density (Zuckier et al., 2000), assuming uniform target distribution on the cell surface, which is not always the case (Wehrman et al., 2006; Byrne et al., 2020). Similarly, when antibody concentrations are low relative to the target, most target molecules on the cell surface are unoccupied and available for antibody binding, assuming little competition from endogenous ligand. A high proportion of antibodies at the cell surface will be bivalently bound under these conditions. In contrast, monovalent bound antibodies will become increasingly prevalent at relatively high antibody concentrations, as antibodies must compete for the free target. These conditions favor monovalent binding and diminishing increases in bivalent engagement with increasing antibody concentration. De Michele et al., suggest antibody size also plays a role in promoting bivalent binding by keeping neighboring molecules at a distance through steric interactions thus ensuring targets within reach of the antibody’s free arm are unoccupied (De Michele et al., 2016).

Work by Bondza et al. demonstrates the influence of free antibody concentration on bivalent binding stability (Bondza et al., 2020). Increasing free antibody concentrations contribute to an increased apparent k_off_, (i.e., reduced binding stability) for monovalent-bound complexes because bivalent binding events must compete with free antibodies for unoccupied targets. In their study, the apparent k_off_ for both rituximab and obinutuzumab differed approximately threefold in tested concentration ranges despite similar antibody affinities. The authors posited that obinutuzumab’s increased k_off_, relative to rituximab, led to more dynamic bivalent target binding than for rituximab, demonstrating an important point: antibodies with similar affinities can display significant differences in their predominant binding mode depending on the relative magnitude of their k_on_ and k_off_. The implications for these concentration-binding-mode relationships are varying bivalent-monovalent ratios of bound antibody on cells/tissues depending on the relative concentration of antibody and target and antibody-target binding characteristics. If bivalent binding stability is advantageous, engineered reductions in k_off_ may be used to promote increased bivalent binding.

### Bivalent Binding and Antibody Selectivity

**FIGURE 3:**
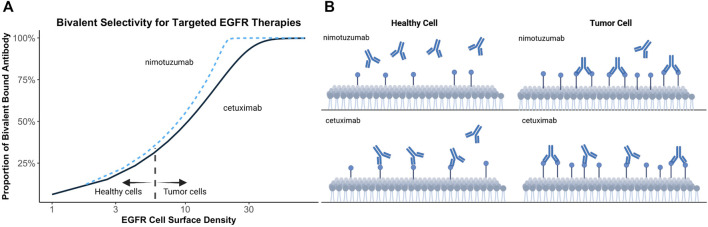
Simulated relationship between target expression and bivalent selectivity. **(A)** Simulation of select EGFR targeted therapies, nimotuzumab (K_D_ = 2.1 × 10^−8^ mol/L; k_on_ = 5.2 × 10^4^ (s mol/L)^−1^; k_off_ = 1.1 × 10^−3^ s^−1^) and cetuximab (K_D_ = 1.8 × 10^−9^ mol/L; k_on_ = 3.1 × 10^6^ (s mol/L)^−1^; k_off_ = 5.8 × 10^−3^ s^−1^) between EGFR expression and maximum proportion of bivalent complex formed. **(B)** Low affinity of nimotuzumab relative to cetuximab prevents accumulation of antibody on healthy cells. Increasing target density promotes bivalent binding and retention of antibody on tumor cells.

Bivalent binding can be leveraged to facilitate antibody selectivity for cells upregulating therapeutic targets. Increasing antibody k_off_ has been recognized as a strategy to promote selective binding to cells upregulating targets, such as HER2 (Slaga et al., 2018). Bivalent binding on disease-associated tissue is promoted by increased target density, avoiding exceedingly high local antibody concentrations, improved cross-arm binding efficiency, and a rebinding-promoting microenvironment. Increased selectivity for disease-associated cells, has also been proposed to explain the differing toxicity profiles of targeted EGFR therapies (Garrido et al., 2011). EGFR is commonly upregulated in human epithelial cancers and is present in healthy tissues (London and Gallo, 2020). Targeted EGFR therapies often demonstrate toxicity associated with on-target off-tumor target binding (Lacouture, 2006; Izzedine et al., 2010). Garrido et al. postulated that nimotuzumab demonstrates a reduced adverse effect profile relative to other EGFR-targeting therapies, such as cetuximab, due to its intermediate affinity for EGFR (Garrido et al., 2011). Monovalent binding of nimotuzumab was prevalent but not efficient to elicit pharmacological actions in cells with low EGFR expressions. Conversely, monovalent cetuximab binding was efficient to trigger pharmacological actions at all examined EGFR densities. This theory may explain why a ten-fold reduction in EGFR affinity of nimotuzumab compared to cetuximab leads to selective binding in tumor tissue while sparing healthy tissues, thereby reducing adverse effects (Garrido et al., 2011). Simulations in Figure 3 demonstrate the relationship between kinetic rate constants and antibody selectivity for select EGFR targeted therapies, cetuximab and nimotuzumab. The steep slope of nimotuzumab with increasing cell surface target density in Figure 3 demonstrates a sharply increasing proportion of bivalent bound antibody with increasing target concentrations due to an intermediate affinity promoting greater selectivity.

### Bispecific Antibodies

As of January 2022, only four bispecific antibody (BsA) products have been approved. However, over 85 bispecific agents were in clinical development in 2019 (Labrijn et al., 2019) suggesting a potential influx of BsA formats in coming years. BsA can be used to bind two targets on the same cell (cis-) or different cells (trans-, i.e., bridge two cells). The ability of BsA to modulate multiple targets may prove advantageous for addressing multifactorial diseases, such as cancer, where target pathway dysregulation, upregulation of alternative pathways, and crosstalk between pathways can lead to treatment resistance (Wu et al., 2015; Thakur et al., 2018). For targets on the same cell, much of the antibody-transmembrane interaction material above applies to BsA; however, antibody affinity must be optimized concerning two targets (Staflin et al., 2020). When density of the therapeutic target is low, an anchoring strategy may be used if other ligands on the cell surface are highly expressed relative to the target. Antibody selectivity can be increased up to 100-fold (Harms et al., 2014) through this strategy if relative concentration conditions between the two targets are met. When expressed in equal proportions, or the anchor target is under-expressed relative to therapeutic target, a bispecific anchor strategy provides only slight advantage over two monoclonal antibodies (Harms et al., 2014). Provided concentration conditions are met, this strategy can improve the selectivity and specificity of the antibody for disease-specific cells, thereby reducing on-target off-site adverse effects. Grugan et al. demonstrate use of an anti-EGFR/c-Met BsA, amivantamab, toward modulating multiple cell surface targets and show binding of one of the two targets is critical to Fc effector function engagement (Grugan et al., 2017).

**FIGURE 4:**
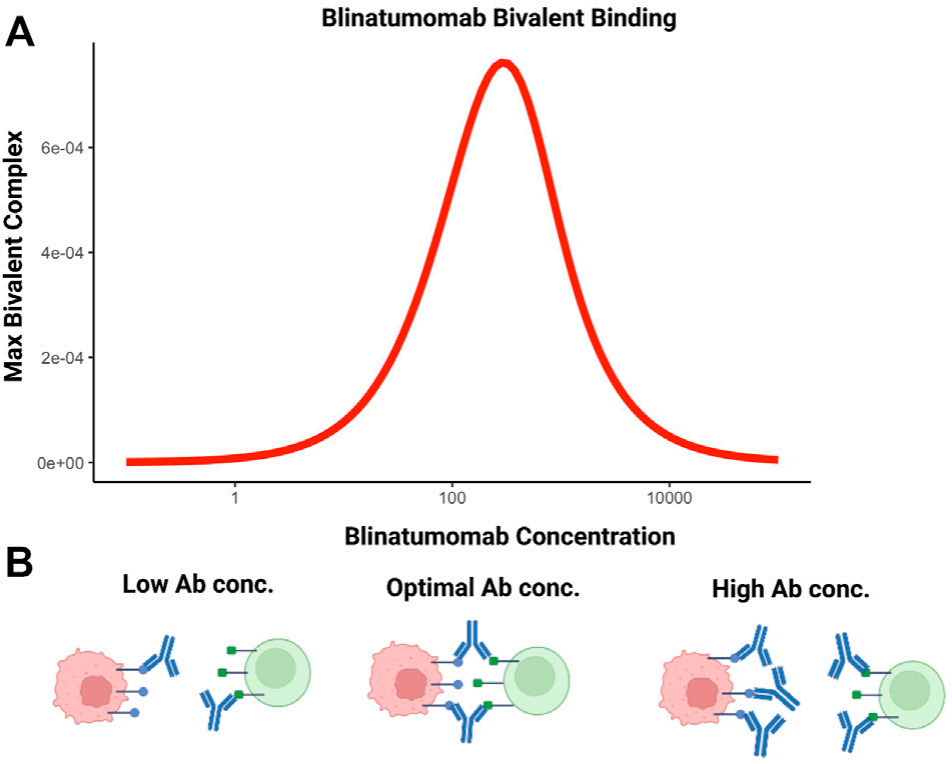
The bell-shaped concentration-effect curves for bispecific T-cell engaging antibodies. **(A)** Simulated relationship between blinatumomab concentration and maximum amount of bivalent complex formed. **(B)** At suboptimal concentrations of bispecific antibodies, bivalent complex formation is limited by the number of bispecific molecules. At optimal concentrations, the number of bivalent complexes formed is maximized. Above the optimal concentration range, target saturation leads to inefficient crosslinking and bivalent complex formation is limited. This condition reduces cell-cell engagement and, potentially, therapeutic efficacy.

BsA can also be used to facilitate cell-cell interactions. For example, blinatumomab, the first in class bispecific T-cell engager molecule is used to promote interaction between T cells and CD19-expressing tumor cells (Einsele et al., 2020). Efficacy of this molecule is based on maximizing the number of bivalent bound complexes; achieved through optimal antibody concentration. The relationship between maximum number of bivalent complexes and increasing antibody concentration is demonstrated by a bell-shape (Betts et al., 2019; Schropp et al., 2019). This phenomenon is attributable to target saturation at higher antibody concentrations. Increasing monovalent complexes compete for targets available for crosslinking, interfering with bivalent complex formation and is depicted in Figure 4.

When used to mediate a bridging strategy between a ligand and receptor attached to cell surfaces, the reaction kinetics will be much different compared to the two species in solution. In this situation, bivalent binding rates are not reflective of interaction affinities, but more generally, the relative rates of cross-linking and intercellular encounter since only adjacent cells can facilitate bivalent binding. Mathematical modeling and experimental interpretation of these reactions is complicated by quantifying the likelihood of cell-cell interaction and potential for additional molecular interactions (e.g., carbohydrates, lectins) between cells contributing to bond avidity (Bell, 1978).

## Conclusion

The number of antibodies and other protein-based therapeutics on the market is increasing rapidly (Kaplon et al., 2020). Despite improved success rates relative to small molecule drugs, the full potential of these molecules will be further realized through rigorous characterization of their *in vivo* target engagement. Additionally, identifying lead drug candidates with optimal target engagement within the tissue/cellular context is paramount to minimizing futile resource allocation in drug development programs. Extensive evidence indicates that the engagement dynamics for antibody-target interactions in living systems differ considerably from that observed *in vitro*. Insight into how the native microenvironment and local physiology influence antibody-target interactions could improve preclinical evaluation, lead optimization, and translation of preclinical candidates to clinical development. Notable takeaways from this work include 1) SPR technologies can serve as a rational basis for antibody screening, but affinity estimates should be used with caution in modeling and simulations depicting target engagement; 2) implementation of local tissue/cellular microenvironment and physiology in preclinical antibody-target engagement models could improve our understanding of *in vivo* antibody-target interactions; 3) antibody physical characteristics, microenvironment, and antibody-target interactions influence the predominant antibody binding mode and can be leveraged to modulate antibody selectivity, distribution, and effector function. Here we briefly reviewed how the interplay between physiological factors and the kinetics of association/dissociation for an antibody-target interaction can influence their engagement *in vivo*. We hope to draw attention to the knowledge gap surrounding the characterization of antibody-target interactions in living systems and demonstrate the relevance of this information to preclinical candidate selection and optimization processes.
